# Nurses' practice concerning sedation during weaning from mechanical ventilation

**DOI:** 10.1186/2197-425X-3-S1-A925

**Published:** 2015-10-01

**Authors:** M Borkowska, S Labeau, S Blot

**Affiliations:** Ghent University, Ghent, Belgium; University College Ghent, Ghent, Belgium

## Introduction

Sedation management during weaning from mechanical ventilation is an important task for ICU nurses. Nurse-led sedation protocols have a favorable impact on weaning outcome [1, 2].

## Objectives

To identify sedation practices among ICU nurses and to determine barriers towards use of sedation protocols.

## Methods

A cross-sectional, self-administrated survey was developed. Consensus on content validity was achieved through a Delphi procedure among experts. The survey was distributed and collected during the annual congress of the Flemish Society of Critical Care Nurses (Dec. 2014).

## Results

423 attendants completed the survey (response rate: 66%) of which 342 ICU nurses were included for further analysis. These are employed in general (73%) or university hospitals (27%) and are working in mixed (66%), surgical (18%) or medical (13%) ICUs.

Short working agents are most frequently chosen for analgo-sedation. A majority of respondents administer sedatives in continuous infusion with bolus doses (81%). Less than half of nurses have a sedation protocol (44%) but only minority consistently uses it (8%). Nurses in university hospitals reported higher availability of sedation protocols compared to general hospitals (72% vs. 42%, p < 0,001). Patient-targeted protocols are most available (53%). They are mostly developed particularly by MDs (79%) and nurses (52%). Level of sedation is generally evaluated per 2 hrs (56%) and with use of a RASS scale (59%). Daily interruption of sedation (DIS) is applied variably (never: 27%, rarely: 54%, mostly 14%, always: 2%) and usually to evaluate the neurological status (86%) and to shorten the duration of ventilation (44%). 78% of nurses report not to apply DIS at night. Patients' discomfort is the most important barrier to execute DIS (49%). Nearly half of respondents pointed out respiratory deterioration is the greatest concern during DIS (47%). For 40% of nurses it is risk of self-extubation and removal of the catheters. Agitation/confusion (66%) and lack of comfort and pain (46%) are the most frequently reported problems associated with sedation reduction.

## Conclusions

There appears to be a considerable discrepancy between recommendations and sedation practice. This data demonstrate room for quality improvement initiatives.

## References

[1] Arias-Rivera S (2008). Crit Care Med.

[2] McGrane S, Pandharipande PP (2012). Minerva Anestesiol.

